# Diverse *PAX6* variants in three Chinese families with congenital aniridia: genotype–phenotype correlations and diagnostic implications

**DOI:** 10.3389/fmed.2026.1978944

**Published:** 2026-10-21

**Authors:** Wen-Jing Ku, Tian-Ying Wei, Jia-Lin Zhang, Ming-Xian Kang, Gang Yu, Jia-En Liu, Jing Zhang, Hua-Ying Hu, Feng Zhang

**Affiliations:** 1 Beijing Meihe Eye Clinic, Beijing Future Children’s Hospital, Beijing, China; 2 Beijing Jiaen Hospital, Heen Life Medical Research Institute, Beijing, China; 3 Clinical College of Chengde Medical University, Chengde, Hebei, China; 4 Prenatal Diagnosis Center, Shijiazhuang Obstetrics and Gynecology Hospital, Hebei Key Laboratory of Maternal and Fetal Medicine, Shijiazhuang, Hebei, China; 5 Medical Innovation Research Division of Chinese PLA General Hospital, Beijing, China

**Keywords:** array comparative genomic hybridization, congenital aniridia, copy number variant, frameshift variant, *PAX6*, whole-exome sequencing

## Abstract

**Purpose:**

To report three families with congenital aniridia harboring different types of *PAX6* variants, analyze their genetic characteristics and genotype–phenotype correlations, and explore the value of a multi-technique combined detection strategy in the molecular diagnosis of aniridia.

**Materials and methods:**

Systematic ophthalmic examinations were performed on the probands and their family members from three aniridia families. Genetic testing was conducted using multiple techniques including high-resolution chromosome karyotyping, array comparative genomic hybridization, whole-exome sequencing, Sanger sequencing, and quantitative real-time polymerase chain reaction. Variant pathogenicity was assessed according to the American College of Medical Genetics and Genomics/Clinical Genome Resource guidelines.

**Results:**

Three pathogenic or likely pathogenic variants were identified in the respective families: Family 1 harbored a heterozygous deletion at 11p13, arr[GRCh37] 11p13(31037165_34227327) × 1 (∼3.19 megabases), encompassing *PAX6*, *WT1*, and several other genes, which occurred *de novo*; Family 2 harbored a heterozygous frameshift variant in *PAX6*, c.1234del (p.Ser412ProfsTer127) on the current MANE Select transcript NM_001368894.2, inherited maternally; Family 3 harbored a *de novo* large deletion at 11p14.1p13, seq[GRCh37] del(11)(p14.1p13) NC_000011.9:g.30352495_33350179del (∼2.998 megabases), also encompassing *PAX6*, *WT1*, and other genes. The two copy number variants were classified as pathogenic and the frameshift variant as likely pathogenic according to the American College of Medical Genetics and Genomics/Clinical Genome Resource standards.

**Conclusions:**

This study reports three different types of pathogenic or likely pathogenic *PAX6* variants, enriching the variant spectrum of aniridia. The combined application of array comparative genomic hybridization, whole-exome sequencing, and Sanger sequencing can significantly improve the molecular diagnostic rate of aniridia. The deletion regions in Families 1 and 3 both encompass the *WT1* gene, meeting the molecular diagnostic criteria for WAGR syndrome. Notably, the proband of Family 3 had been diagnosed with Wilms tumor prior to presentation, underscoring the importance of precise genetic diagnosis for clinical management and genetic counseling.

## Introduction

Congenital aniridia (OMIM #106210) is a rare inherited ocular disorder characterized primarily by iris hypoplasia or aplasia, with an estimated prevalence of 1/64,000 to 1/96,000 (1). The condition is not merely an isolated iris abnormality but rather a panocular disease affecting the entire eye, frequently accompanied by corneal pathology (aniridia-associated keratopathy, AKP), cataract, glaucoma, foveal hypoplasia, and nystagmus, severely impairing visual function (2, 3).

The *PAX6* gene (paired box gene 6, OMIM *607108) is located on chromosome 11p13 and encodes a highly conserved paired-box transcription factor that serves as a master regulator of eye development, playing an indispensable role in the development of the lens, cornea, retina, and iris (4). The *PAX6* gene comprises 14 exons, including the alternatively spliced exon 5a, and encodes a protein containing a paired box domain, a homeodomain, and a proline-serine-threonine (PST) transactivation domain (5). *PAX6* haploinsufficiency is the principal pathogenic mechanism underlying congenital aniridia, with heterozygous loss-of-function *PAX6* variants detectable in approximately 80%–90% of patients (6).

To date, more than 500 pathogenic *PAX6* variants have been reported, encompassing nonsense variants, frameshift variants, splice-site variants, missense variants, and copy number variants (CNVs) (5). Among these, CNVs—including intragenic deletions, whole-gene deletions, and distal deletions encompassing *PAX6* regulatory regions—account for approximately 10%–20% of all pathogenic variants (6). When the 11p13 deletion region simultaneously encompasses the *WT1* gene, patients may present with WAGR syndrome (Wilms tumor, aniridia, genitourinary anomalies, and intellectual disability; OMIM #194072), carrying a high risk of developing Wilms tumor (7).

Currently, diverse molecular diagnostic methods are available for aniridia, including Sanger sequencing, multiplex ligation-dependent probe amplification (MLPA), chromosomal microarray, and next-generation sequencing (NGS), with varying detection capabilities for different variant types (8). Multi-technique combined approaches can significantly improve the molecular diagnostic rate; however, studies systematically reporting and comparing multiple variant types in the Chinese population remain limited (9, 10).

Despite these advances, several challenges remain in the diagnosis and clinical management of congenital aniridia. First, phenotypic heterogeneity—including milder or atypical presentations such as partial iris hypoplasia or iris coloboma—can delay clinical recognition and appropriate molecular testing. Second, the diversity of pathogenic variant types, spanning single-nucleotide variants, small insertions/deletions, splice-site variants, and large copy number variants (CNVs) affecting *PAX6* or its regulatory regions, means that no single detection technique is sufficient, and the choice and sequence of tests directly determine the diagnostic yield. Third, when the deletion co-involves *WT1*, early molecular diagnosis is critical to initiate Wilms tumor surveillance within the window of maximal tumor risk. Finally, precise identification of the familial variant underpins reproductive counseling, carrier detection, and prenatal diagnosis. Addressing these challenges requires systematic, multi-technique genetic evaluation, as illustrated in this study.

This study reports three families with congenital aniridia harboring different types of pathogenic or likely pathogenic *PAX6* variants: a ∼3.19 Mb heterozygous deletion at 11p13 (Family 1), a *PAX6* frameshift variant c.1234del (NM_001368894.2) (Family 2), and a ∼2.998 Mb *de novo* large deletion at 11p14.1p13 (Family 3). Through the integrated application of high-resolution chromosome karyotyping, aCGH, WES, and qPCR, systematic genetic analyses were performed for all three families, with the aim of enriching the *PAX6* variant spectrum and exploring the utility of different detection strategies in the precise diagnosis of aniridia. Specifically, this report (i) provides a side-by-side illustration of aCGH-based and WES read-depth-based CNV detection within a single diagnostic framework, (ii) demonstrates how a WES-based CNV finding directly informed clinical management through Wilms tumor surveillance in a child with WAGR spectrum disorder, and (iii) illustrates the immediate reproductive utility of a precise molecular diagnosis through prenatal diagnosis in Family 2.

## Materials and methods

### Study subjects

This study enrolled three families with congenital aniridia (Families 1, 2, and 3), comprising a total of 11 family members (3 probands, 7 other family members, and 1 fetus undergoing prenatal diagnosis). All participants provided written informed consent; for minors, consent was obtained from their legal guardians. The study was approved by the Ethics Committee of Beijing Jiaen Hospital (approval no.: BJH-2026004; approval date: June 21, 2026) and conducted in accordance with the principles of the Declaration of Helsinki. Written informed consent for the publication of clinical data and ophthalmic images was obtained from all participants or their legal guardians.

### Sample collection and processing

EDTA-anticoagulated peripheral venous blood samples were collected from the probands and their family members for whole-exome sequencing, aCGH, Sanger sequencing, and qPCR validation. Heparin-anticoagulated peripheral blood samples were collected for high-resolution chromosome karyotyping. Amniotic fluid samples were obtained from the fetus (2.4) undergoing prenatal diagnosis for targeted Sanger sequencing of the c.1234del variant.

### Clinical evaluation

All probands underwent comprehensive ophthalmic examinations, including best-corrected visual acuity (BCVA), slit-lamp biomicroscopy, color fundus photography, ocular B-scan ultrasonography, optical coherence tomography (OCT), intraocular pressure measurement, and axial length biometry. The clinical diagnosis was established based on the characteristic findings of iris hypoplasia or aplasia, in conjunction with the aforementioned ancillary examinations.

### Chromosomal microarray analysis (aCGH)

Genomic DNA was extracted from peripheral blood using QIAGEN (Hilden, Germany) reagents. DNA concentration was measured using a Thermo Fisher Scientific (Waltham, MA, USA) NanoDrop 2000 and adjusted to 50 ng/*μ*l. Genome-wide CNV detection was performed using the Affymetrix (Santa Clara, CA, USA) CytoScan 750 K Array, with concurrent analysis of loss of heterozygosity (LOH) and uniparental disomy. Following the manufacturer's standard protocols, genomic DNA was subjected to digestion, ligation, amplification, purification, fragmentation, and labeling. The labeled samples were hybridized to the array at 50 °C and 60 rpm for 16–18 h. After washing, staining, and scanning, data analysis was performed using Chromosome Analysis Suite (ChAS) software with the human genome GRCh37 as the reference sequence, at a resolution of 200 kb (11).

### High-resolution chromosome karyotyping

Following the laboratory standards of the American College of Medical Genetics and Genomics, heparin-anticoagulated blood (0.45 mL) was inoculated into cell culture medium within a biosafety cabinet and cultured at 37 °C for 72 h. Harvesting, banding, and slide preparation were performed according to standard operating procedures. Metaphase chromosome spreads were scanned and captured using a Leica (Wetzlar, Germany) GSL-120 automated cytogenetics workstation, and karyotype analysis was assisted by CytoVision software with manual verification. Chromosome-level abnormalities were assessed based on the analytical results.

### Whole-exome sequencing (WES)

Genomic sequencing was performed on the Illumina (San Diego, CA, USA) NovaSeq 6000 platform using the IDT (Coralville, IA, USA) xGen Exome Research Panel v2.0 for capture and library preparation, with a paired-end 150 bp (PE150) sequencing strategy. Raw sequencing reads (quality level %Q30 > 89%) were aligned to the human reference genome (hg19/GRCh37) using the Burrows-Wheeler Aligner (BWA), and PCR duplicates were removed using Picard v1.57. Identified variants were annotated against genetic variant databases (including ClinVar and gnomAD) and classified according to the ACMG/AMP guidelines (12). Pathogenicity was predicted using SIFT, PolyPhen-2, and MutationTaster2. Candidate variants were validated by Sanger sequencing (ABI 3730xl), with co-segregation analysis performed in available family members.

CNV analysis from WES data

Read-depth-based CNV detection from WES data was performed using ML-ExonCNV v1.0.0 (13), an XGBoost multi-expert ensemble tool for exon-level CNV detection from WES data. The WES data of the proband were generated using the IDT xGen Exome Research Panel v2.0 capture system (target-region coverage 99.80%; >20× coverage 99.30%; Q30 = 0.98). A control library was built from deduplicated BAM files of WES control samples processed within the same experimental system (identical capture probes and standard operating procedure). Following the standard workflow of the software, read-count profiles over 200-bp virtual target regions (MANE Select CDS) were used, and at least 10 control samples most similar to the test sample were automatically selected by Pearson correlation analysis, followed by loess regression-based GC correction and *Z*-testing, with population-frequency filtering against an in-house CNV database. CNVs were reported according to the laboratory reporting thresholds (CNV > 500 kb, or at least three consecutive exons with sufficient coverage). Candidate CNVs were reviewed as normalized read-depth plots, and all CNVs were independently validated by qPCR as described below.

### qPCR and sanger sequencing validation

Primers were designed based on gene sequences from the Ensembl genome browser using the NCBI Primer-BLAST tool (see Supplementary Table S1). qPCR was performed using TB Green Premix Ex Taq GC (Perfect Real Time) reagents on an Applied Biosystems (Foster City, CA, USA) 7500 Fast DX analyzer to verify CNV carrier status in the probands and family members. *HBB* was used as the reference gene, with the mean values from three healthy individuals as the reference. Data were analyzed using the 2^-*ΔΔ*Ct^ method.

For Sanger sequencing, target sequences were amplified using PrimeSTAR Max DNA Polymerase (TaKaRa, Kusatsu, Japan); primer sequences and amplification conditions are provided in Supplementary Table S2. PCR products were purified using the QIAquick PCR Purification Kit (QIAGEN). Sanger sequencing was performed using the BigDye Terminator v3.1 Cycle Sequencing Kit (Applied Biosystems) on an Applied Biosystems 3730XL Genetic Analyzer (Thermo Fisher).

## Results

### Family 1: Detection of an 11p13 heterozygous deletion

#### Clinical presentation

The proband of Family 1 (1.3) was a female who presented with poor bilateral vision. Slit-lamp examination revealed bilateral iris absence with punctate posterior synechiae and nystagmus (Figure 1A). Ocular B-scan ultrasonography demonstrated no iris echoes within the anterior chamber, with iris stumps detectable at the iridocorneal angle. Fundus photography showed an essentially normal bilateral fundus. Biometry revealed an axial length of 24.46 mm in the right eye and 24.17 mm in the left eye. Ophthalmic examinations of her father (1.1), mother (1.2), and younger sister (1.4) revealed no significant abnormalities.

**Figure 1:**
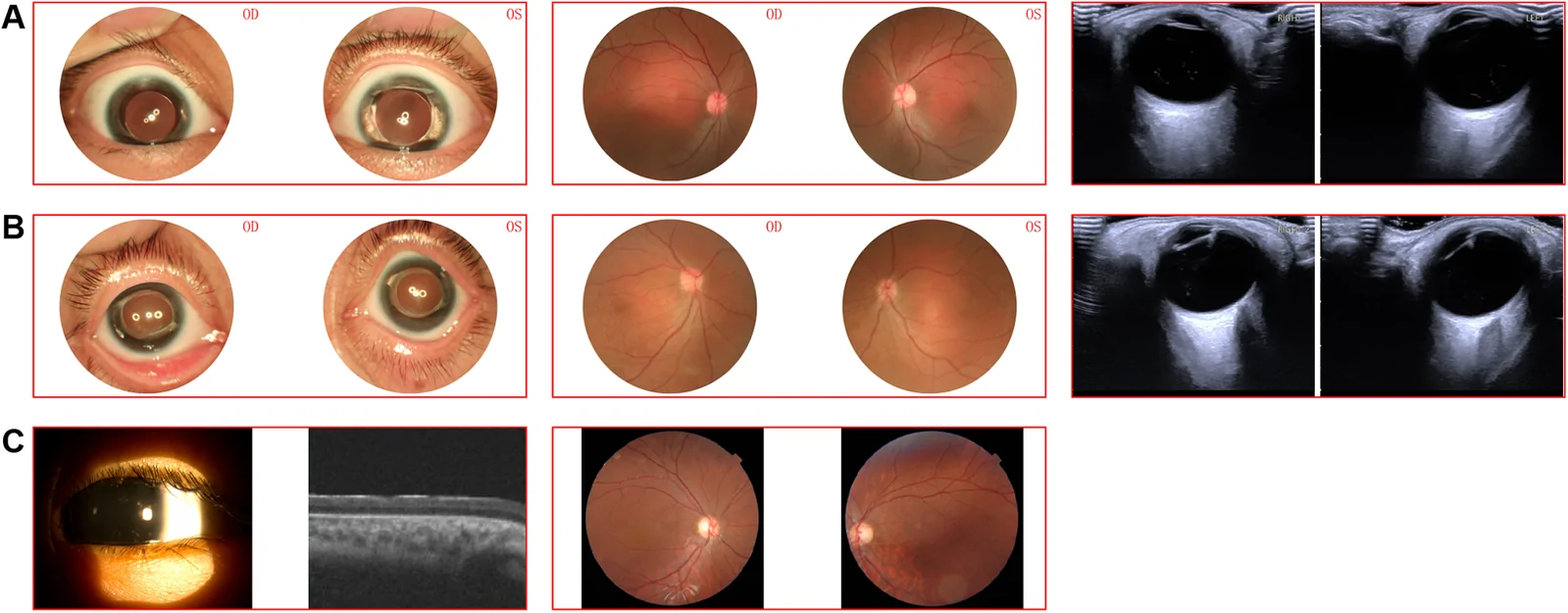
Ophthalmic examination results of the three probands. **(A)** Ophthalmic examination of the Family 1 proband (1.3): left panel, slit-lamp photograph showing bilateral iris absence with punctate posterior synechiae; middle panel, bilateral fundus photographs, essentially normal; right panel, ocular B-scan ultrasonography showing no iris echoes within the anterior chamber, with iris stumps detectable at the iridocorneal angle. **(B)** Ophthalmic examination of the Family 2 proband (2.3): left panel, slit-lamp photograph showing bilateral iris absence with anterior subcapsular punctate opacities of the lens; middle panel, bilateral fundus photographs, essentially normal; right panel, ocular B-scan ultrasonography showing no iris echoes within the anterior chamber, with short band-like iris stumps attached to the ciliary body at the iridocorneal angle. **(C)** Ophthalmic examination of the Family 3 proband (3.3): left panel, slit-lamp photograph of the left eye and macular OCT showing iris absence and foveal hypoplasia; right panel, bilateral fundus photographs showing mild tessellated appearance in the posterior pole, with an inconspicuous foveal reflex in the left macula. OD: right eye; OS: left eye.

#### Comprehensive genetic testing

The pedigree of Family 1 showed that the proband (1.3) was the sole affected individual, with both parents (1.1, 1.2) and the younger sister (1.4) being phenotypically normal, suggesting a *de novo* variant (Figure 2A).

**Figure 2:**
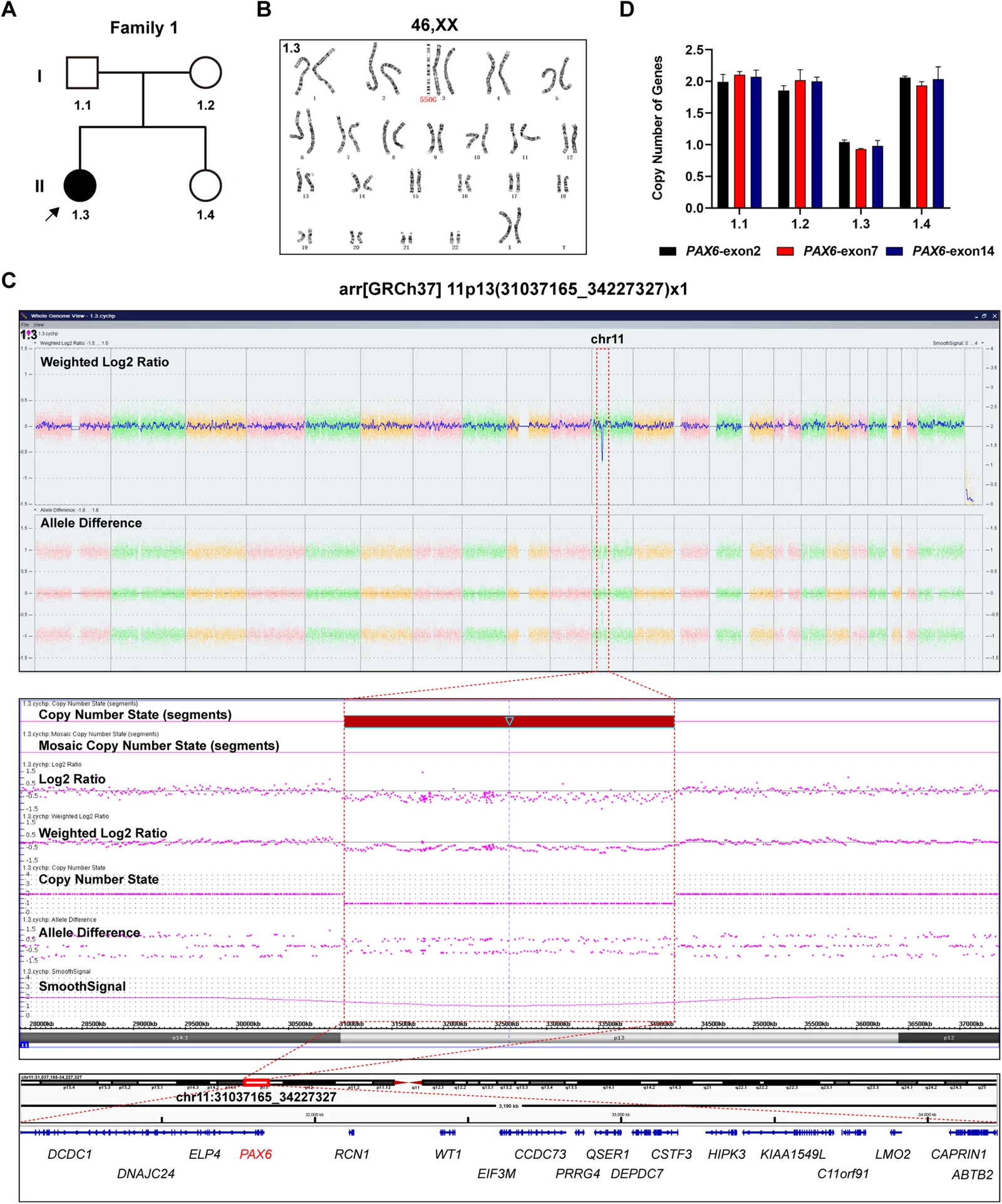
Genetic testing results of family 1. **(A)** Pedigree of Family 1; ▪/● indicate affected individuals, □/○ indicate phenotypically normal individuals, and the arrow indicates the proband (1.3). **(B)** High-resolution chromosome karyotyping of the proband (1.3): 46,XX, with no apparent abnormalities. **(C)** aCGH results: upper panel, genome-wide CNV overview; lower panel, magnified view of the 11p13 region, with the red box indicating the ∼3.19 Mb heterozygous deletion [arr[GRCh37] 11p13(31037165_34227327) × 1]; gene annotation shows that the deletion region encompasses *PAX6* and other genes. **(D)** qPCR validation results: relative copy numbers of *PAX6* exons 2, 7, and 14 in the proband (1.3) are approximately 1.0, while copy numbers in the father (1.1), mother (1.2), and younger sister (1.4) are normal.

High-resolution chromosome karyotyping revealed a karyotype of 46,XX in the proband (1.3), with no apparent numerical or structural chromosomal abnormalities (Figure 2B). High-resolution karyotyping (550–700 band resolution) failed to detect the microdeletion at the 11p13 region.

aCGH analysis identified a ∼3.19 Mb heterozygous deletion at the 11p13 region in the proband (1.3): arr[GRCh37] 11p13(31037165_34227327) × 1 (Figure 2C). The deletion region encompassed the entire *PAX6* gene as well as *WT1*, *ELP4*, *RCN1*, *CCDC73*, and several other genes. Because the deletion region encompassed both *PAX6* and *WT1*, the proband met the molecular diagnostic criteria for WAGR syndrome. According to the ACMG/ClinGen CNV interpretation standards (11), this deletion was classified as pathogenic (evidence: 2A + 5A + 5H).

qPCR validation showed that the relative copy numbers of *PAX6* exons 2, 7, and 14 in the proband (1.3) were approximately 1.0, indicating a single-copy deletion. qPCR results for the father (1.1), mother (1.2), and younger sister (1.4) were normal, confirming the deletion as *de novo* (Figure 2D).

### Family 2: Detection of a heterozygous *PAX6* frameshift variant (NM_001368894.2:c.1234del)

#### Clinical presentation

The proband of Family 2 (2.3) was a female with a history of poor bilateral vision and photophobia since childhood, accompanied by nystagmus. Slit-lamp examination revealed bilateral iris absence with anterior subcapsular punctate opacities of the lens (Figure 1B). Ocular B-scan ultrasonography demonstrated no iris echoes within the anterior chamber, with short band-like iris stumps attached to the ciliary body detectable at the iridocorneal angle. Fundus photography showed an essentially normal bilateral fundus. The proband's mother (2.2) also exhibited bilateral iris hypoplasia, while her father (2.1) had no significant ophthalmic abnormalities, consistent with an autosomal dominant inheritance pattern.

#### Comprehensive genetic testing

The pedigree of Family 2 demonstrated an autosomal dominant inheritance pattern, with the proband (2.3) and her mother (2.2) being affected, the father (2.1) phenotypically normal, and 2.4 being a fetus undergoing prenatal diagnosis (amniotic fluid sample, sex unknown) (Figure 3A).

**Figure 3:**
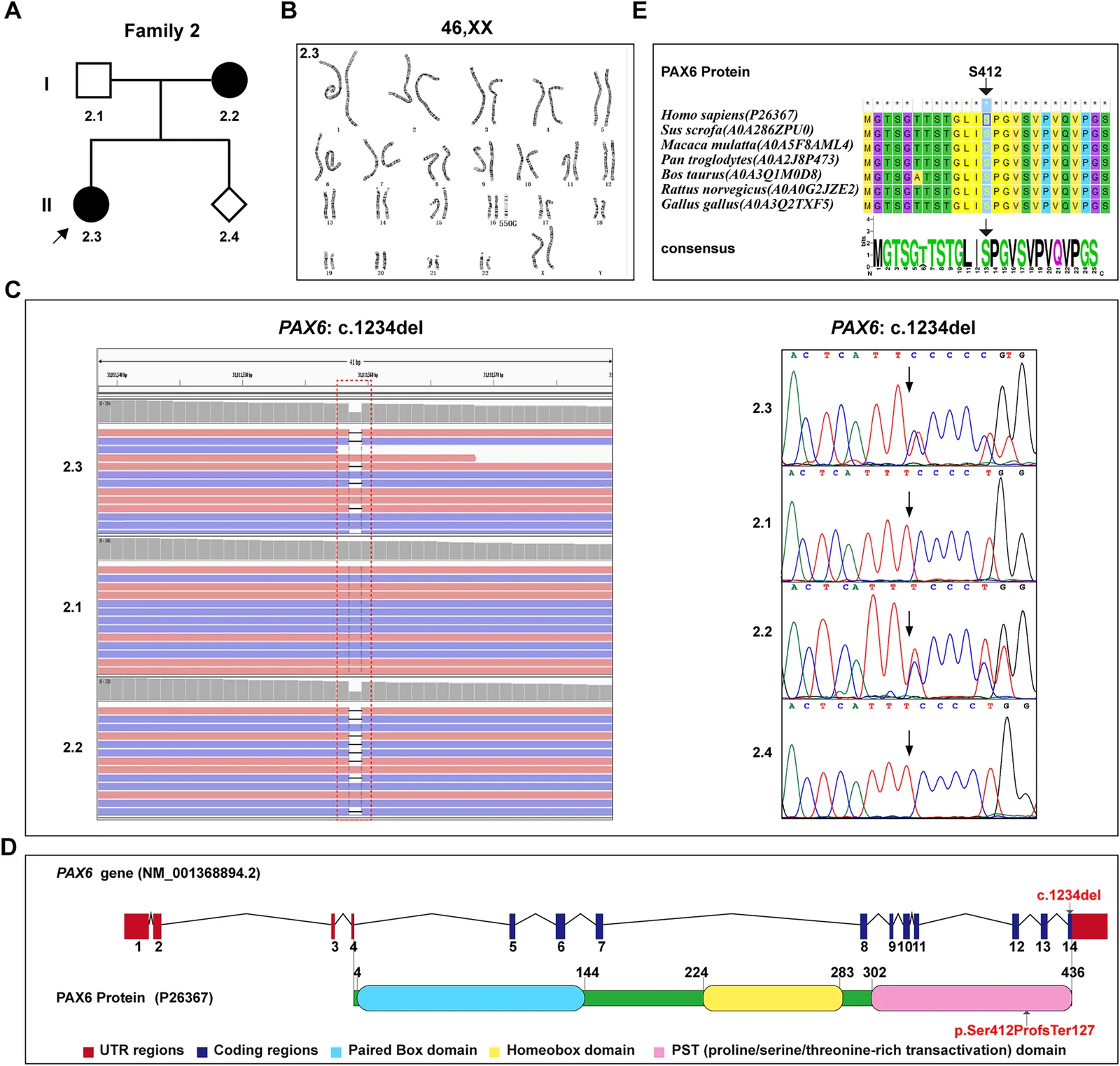
Genetic testing results of family 2. **(A)** Pedigree of Family 2; ▪/● indicate affected individuals, □/○ indicate phenotypically normal individuals, the arrow indicates the proband (2.3), and ◇ indicates the fetus (2.4) of unknown sex undergoing prenatal diagnosis. **(B)** Chromosome karyotyping of the proband (2.3): 46,XX, with no apparent abnormalities. **(C)** WES read-depth visualization (Integrative Genomics Viewer; GRCh37 coordinates) of the *PAX6* c.1234del region in the proband (2.3), her father (2.1), and her mother (2.2): the red dashed box indicates the heterozygous deletion, which is present in the proband and her mother (coverage dip with split reads) and absent in her father. Sanger sequencing confirmed that the proband (2.3) and her mother (2.2) carry the *PAX6* c.1234del heterozygous variant (arrow indicates the deletion site), while the father (2.1) and fetus (2.4) have normal sequencing results. **(D)** Schematic diagram of the *PAX6* gene (NM_001368894.2) and protein (P26367) structure, showing exons 1–14 and the protein domains (Paired Box domain, Homeobox domain, and PST transactivation domain), with the arrow indicating the c.1234del variant position in the terminal coding exon (exon 14; p.Ser412ProfsTer127). **(E)** Cross-species conservation analysis showing that the S412 residue is highly conserved across humans, chimpanzees, rhesus macaques, pigs, cattle, rats, and chickens.

High-resolution chromosome karyotyping revealed a karyotype of 46,XX in the proband (2.3), with no apparent numerical or structural chromosomal abnormalities (Figure 3B).

WES identified a heterozygous single-base deletion in exon 14 of *PAX6* in the proband (2.3) (Figure 3C). On the MANE Select transcript (NM_001368894.2; ENST00000640368.2), the variant is described as c.1234del (p.Ser412ProfsTer127), with the genomic description NC_000011.9:g.31811561del on GRCh37 (hg19), the reference build used in this study. Per the HGVS 3′ rule, the deleted base lies within a thymine homopolymer (c.1232–c.1234), and the frameshift begins at serine 412. Because the frameshift is located in the terminal coding exon, it is predicted to escape nonsense-mediated mRNA decay and to encode a C-terminally extended protein of 537 amino acids (wild type, 436). This variant was not recorded in public databases including gnomAD and ClinVar, representing a novel variant.

Sanger sequencing validation confirmed that both the proband (2.3) and her mother (2.2) carried the heterozygous variant, while the father (2.1) did not, confirming maternal inheritance with co-segregation of the variant and the family phenotype. Targeted Sanger sequencing of the amniotic fluid sample obtained for prenatal diagnosis showed that the fetus (2.4) did not carry the variant (Figure 3C).

Structural analysis of the *PAX6* gene and protein showed that the variant is located in exon 14 of the MANE Select transcript (NM_001368894.2), corresponding to the PST transactivation domain region of the protein (Figure 3D). Cross-species conservation analysis demonstrated that the S412 residue is highly conserved across humans, chimpanzees, rhesus macaques, pigs, cattle, rats, and chickens (Figure 3E), suggesting important functional significance of this site in evolution. According to the ACMG sequence variant interpretation standards (12), the ClinGen Sequence Variant Interpretation Working Group recommendations for the PVS1 criterion were applied (14): because the frameshift is located in the terminal exon and is predicted to escape nonsense-mediated mRNA decay, PVS1 was applied at the strong level (PVS1_Strong). Co-segregation evidence (PP1) was not applied, as the variant segregated with the disease in only a single meiosis. Using the point-based classification framework (15), the combined evidence was PVS1_Strong (4 points) + PM2_Supporting (1 point) + PP3 (1 point) + PP4 (1 point) = 7 points, and the variant was therefore classified as likely pathogenic.

### Family 3: Detection of an 11p14.1p13 heterozygous deletion

#### Clinical presentation

The proband of Family 3 (3.3) was a male in whom bilateral iris absence was noted at birth, accompanied by nystagmus and photophobia. Slit-lamp examination revealed iris absence; macular OCT demonstrated foveal hypoplasia in the left eye (Figure 1C). Fundus photography showed a mild tessellated appearance in the posterior pole of both eyes, with an inconspicuous foveal reflex in the left macula. Biometry revealed an axial length of 26.65 mm in the right eye and 26.50 mm in the left eye. Ophthalmic examinations of both parents (3.1, 3.2) revealed no significant abnormalities, suggesting a *de novo* variant. Additionally, the proband exhibited mild intellectual disability and had been diagnosed with Wilms tumor prior to presentation.

#### Comprehensive genetic testing

The pedigree of Family 3 showed that the proband (3.3) was the sole affected individual, with both parents (3.1, 3.2) being phenotypically normal, suggesting a *de novo* variant (Figure 4A).

**Figure 4:**
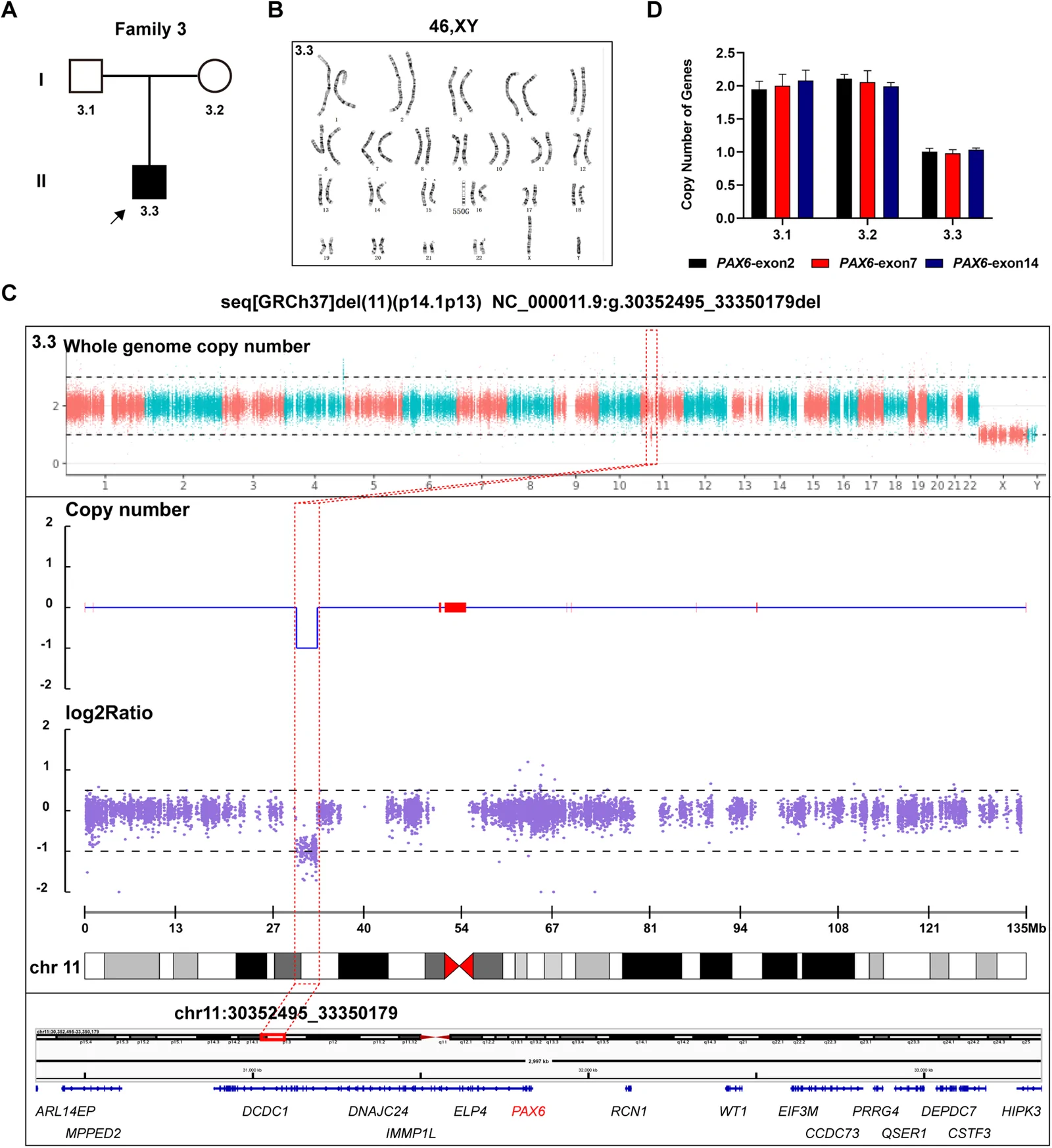
Genetic testing results of family 3. **(A)** Pedigree of Family 3; ▪/● indicate affected individuals, □/○ indicate phenotypically normal individuals, and the arrow indicates the proband (3.3). **(B)** Chromosome karyotyping of the proband (3.3): 46,XY, with no apparent abnormalities. **(C)** WES results: upper panel, genome-wide CNV overview; middle panel, magnified view of chromosome 11; lower panel, gene annotation of the 11p14.1p13 region, with the red box indicating the ∼2.998 Mb heterozygous deletion [seq[GRCh37] del(11)(p14.1p13) NC_000011.9:g.30352495_33350179del]; the deletion region encompasses *PAX6*, *WT1*, and other genes. **(D)** qPCR validation results: relative copy numbers of *PAX6* exons 2, 7, and 14 in the proband (3.3) are approximately 1.0, while copy numbers in the father (3.1) and mother (3.2) are normal, confirming a *de novo* variant.

High-resolution chromosome karyotyping revealed a karyotype of 46,XY in the proband (3.3), with no apparent numerical or structural chromosomal abnormalities (Figure 4B).

Read-depth-based CNV analysis of the WES data (ML-ExonCNV v1.0.0) initially identified a ∼2.998 Mb heterozygous deletion at the 11p14.1p13 region in the proband (3.3): seq[GRCh37] del(11)(p14.1p13) NC_000011.9:g.30352495_33350179del (Figure 4C). Across the deleted interval, the calculated copy number was −1 and the test/control log2 depth ratio clustered at approximately −1.0 (points spanning roughly −0.7 to −1.5), consistent with the theoretical value for a heterozygous deletion, with significant Z-scores, while flanking regions fluctuated around 0; the deleted interval completely encompasses *PAX6* (exons 1–14) and *WT1* (exons 1–10). The copy number state was independently validated by qPCR (see below). The deletion region encompassed the entire *PAX6* gene as well as *WT1* (Wilms tumor 1), *DCDC1*, *DNAJC24*, *ELP4*, *RCN1*, and other genes. Because the deletion region encompassed both *PAX6* and *WT1*, the proband met the molecular diagnostic criteria for WAGR syndrome (7). According to the ACMG/ClinGen CNV interpretation standards (11), this deletion was classified as pathogenic (evidence: 2A + 5A + 5H).

qPCR validation showed that the relative copy numbers of *PAX6* exons 2, 7, and 14 in the proband (3.3) were approximately 1.0, indicating a single-copy deletion. qPCR results for both the father (3.1) and mother (3.2) were normal, confirming the deletion as a *de novo* variant (Figure 4D). The genetic and clinical features of the three families are summarized in Table 1.

**Table 1:** Summary of genetic and clinical features of the three families with congenital aniridia.

Feature	Family 1	Family 2	Family 3
Genetic characteristics			
Variant type	Chromosomal microdeletion (CNV)	Frameshift variant	Chromosomal microdeletion (CNV)
Variant description	arr[GRCh37] 11p13 (31037165_34227327) × 1	NM_001368894.2 (MANE Select): c.1234del (p.Ser412ProfsTer127)	seq[GRCh37] del(11)(p14.1p13) NC_000011.9:g.30352495_33350179del
Deletion size	∼3.19 Mb	1 bp (single-base deletion)	∼2.998 Mb
Inheritance pattern	*De novo*	Autosomal dominant (maternal)	*De novo*
WAGR risk	Yes (includes *WT1*)	No	Yes (includes *WT1*)
Primary detection method	aCGH+qPCR	WES+Sanger sequencing	WES (ML-ExonCNV) + qPCR
ACMG classification	Pathogenic (2A + 5A + 5H)	Likely pathogenic (PVS1_Strong + PM2_Supporting +PP3 + PP4)	Pathogenic (2A + 5A + 5H)
Database record	Not reported	Not reported	Not reported
Ophthalmic manifestations			
Iris abnormality	Bilateral iris absence with punctate posterior synechiae	Bilateral iris absence with anterior subcapsular punctate opacities	Bilateral iris absence
Foveal hypoplasia	Not confirmed	Not confirmed	Yes (foveal hypoplasia confirmed by OCT)
Nystagmus	Yes	Yes	Yes
Lens changes	No significant opacity	Anterior subcapsular punctate opacities	Clear lens
Retinal changes	No significant abnormality	No significant abnormality	Mild tessellated appearance in posterior pole of both eyes
Fundus/B-scan	Fundus largely normal; B-scan shows iris stump at iridocorneal angle	Fundus largely normal; B-scan shows short band-like iris stump attached to ciliary body	Tessellated appearance in posterior pole; left macular foveal reflex inconspicuous
Axial length (mm)	OD 24.46/OS 24.17	Not recorded	OD 26.65/OS 26.50
Systemic manifestations			
Intellectual development	Normal	Normal	Mild delay
Renal abnormalities	Requires monitoring (WAGR risk)	None	Wilms tumor diagnosed
Urogenital abnormalities	Requires evaluation	None	Requires evaluation
Other systemic abnormalities	None	None	None
Clinical management recommendations			
Tumor surveillance	Required (abdominal ultrasound every 3 months until age 8)	Not required	Required (diagnosed; treatment and follow-up needed)
Recurrence risk	Low recurrence risk for parents; 50% risk for offspring of proband	50% risk for offspring	Low recurrence risk for parents; 50% risk for offspring of proband
Multidisciplinary management	Ophthalmology+Nephrology+Oncology+Neurodevelopment	Ophthalmology follow-up	Ophthalmology+Nephrology+Oncology+Neurodevelopment

## Discussion

This study reports three families with congenital aniridia harboring three distinct types of pathogenic or likely pathogenic *PAX6* variants: a ∼3.19 Mb heterozygous deletion at 11p13 (Family 1), a *PAX6* frameshift variant c.1234del (NM_001368894.2) (Family 2), and a ∼2.998 Mb *de novo* large deletion at 11p14.1p13 (Family 3). All three variants were confirmed through multi-technique combined detection and classified as pathogenic (the two copy number variants) or likely pathogenic (the frameshift variant) according to the ACMG/ClinGen standards, further enriching the variant spectrum of *PAX6*-related aniridia.

The transcription factor encoded by *PAX6* plays a central regulatory role in eye development, and *PAX6* haploinsufficiency is the principal pathogenic mechanism of congenital aniridia (4, 6). Both point variants (nonsense, frameshift, and splice-site variants) and large deletions encompassing *PAX6* can reduce the expression of functional *PAX6* protein, leading to developmental abnormalities of the iris, cornea, lens, and macula (5). Although the variant types in the three families in this study differed, all resulted in similar aniridia phenotypes through the *PAX6* haploinsufficiency mechanism, consistent with previous reports (1, 3).

The c.1234del variant identified in Family 2 (NM_001368894.2; p.Ser412ProfsTer127) is a single-base deletion in the terminal coding exon of *PAX6*. Per the HGVS 3′ rule, the frameshift begins at amino acid position 412. Because the frameshift lies in the terminal exon, the variant transcript is predicted to escape nonsense-mediated mRNA decay (NMD); the resulting open reading frame encodes a C-terminally extended protein of 537 amino acids (wild type, 436), in which the C-terminal 25 residues of the PST transactivation domain are replaced by a 126-amino-acid aberrant tail. Disruption of the PST transactivation domain, rather than NMD-mediated haploinsufficiency (6, 16), is therefore the most likely pathogenic mechanism. The S412 residue is highly conserved across humans, chimpanzees, rhesus macaques, pigs, cattle, rats, and chickens, further supporting its functional importance. Previous reviews have shown that *PAX6* frameshift variants are widely distributed across all exons and are consistently associated with typical aniridia phenotypes (5), which is consistent with our findings. This variant was not recorded in public databases, representing a novel pathogenic variant that enriches the *PAX6* frameshift variant spectrum.

Family 1 (∼3.19 Mb deletion) and Family 3 (∼2.998 Mb deletion) both involved large deletions at the 11p13/p14.1p13 region, but differed significantly in deletion scope and clinical implications. The deletion region in Family 1 (31,037,164–34,227,327 bp) encompassed the *WT1* gene, meeting the molecular diagnostic criteria for WAGR syndrome; however, the patient had not yet manifested tumor or intellectual disability at the time of presentation, and the inheritance pattern was *de novo*. Similar 11p13 deletions have been reported in the Chinese population, presenting as isolated aniridia or with mild intellectual disability (17). The deletion region in Family 3 (30,352,495–33,350,179 bp) encompassed both *PAX6* and *WT1*, also meeting the molecular diagnostic criteria for WAGR syndrome. The risk of Wilms tumor in patients with WAGR syndrome is approximately 42.5%–77%, with the majority of tumors occurring before 4 years of age (7, 18). The proband of Family 3 had been diagnosed with Wilms tumor prior to presentation, necessitating oncological treatment and long-term follow-up (7, 19). Furthermore, the deletions in both Family 1 and Family 3 were *de novo*, suggesting a low recurrence risk for the parents, although the possibility of gonadal mosaicism must be considered. Notably, the deletions in both CNV families were detected by aCGH or WES, whereas conventional chromosome karyotyping failed to identify abnormalities in either case, reaffirming the necessity of high-resolution genomic testing in the molecular diagnosis of aniridia (8, 19).

The three families in this study employed different primary detection techniques: Family 1 used aCGH, while Families 2 and 3 used WES, supplemented by chromosome karyotyping, Sanger sequencing, and qPCR for validation and co-segregation analysis. The advantages of this multi-technique combined strategy include: (1) both aCGH and WES can efficiently detect CNVs, allowing flexible selection based on practical considerations; (2) WES offers high sensitivity for detecting point variants and small indels; (3) qPCR enables independent quantitative validation of CNVs and family co-segregation analysis; and (4) Sanger sequencing remains the gold standard for point variant validation. Wang et al. (8) demonstrated that the combined use of NGS and MLPA achieved an overall variant detection rate of 98.3% in Chinese aniridia patients; the multi-technique strategy employed in this study is consistent with this approach, further supporting the value of comprehensive testing protocols in the precise diagnosis of aniridia.

Beyond the individual variant descriptions, several aspects of this work merit emphasis. First, the three families illustrate, within a single diagnostic framework, a direct comparison between aCGH-based and WES read-depth-based CNV detection: both approaches identified the large deletions, while WES additionally detected the single-base frameshift variant, supporting WES-based CNV analysis as a flexible alternative when dedicated array platforms are unavailable. Second, the frameshift variant in Family 2 was annotated on the current MANE Select transcript, providing an updated reference description for this C-terminal-extension allele. Third, Family 3 provides a clinically important lesson: the Wilms tumor diagnosis preceded the molecular confirmation of the underlying 11p14.1p13 deletion, underscoring that molecular diagnosis of aniridia can retrospectively establish tumor risk and should be pursued promptly, including in apparently sporadic cases. Finally, the prenatal diagnosis in Family 2 demonstrates the immediate reproductive utility of identifying the familial variant. Reported *PAX6* variants in patients with congenital aniridia across different populations, together with their phenotypes and detection methods, are summarized in Supplementary Table S3.

All three probands presented with typical aniridia, including iris aplasia or severe hypoplasia and nystagmus. The proband of Family 3 was confirmed by OCT to have foveal hypoplasia, consistent with previously reported manifestations associated with *PAX6* loss-of-function variants.(1, 3, 5) Kit et al. (3) conducted a longitudinal study of 86 patients with *PAX6*-related aniridia and found that patients with deletion-type CNVs tended to have more severe anterior segment manifestations than those with point variants; however, the limited sample size in this study precludes systematic comparison of phenotypic severity across the three variant types. Additionally, the proband of Family 3 exhibited mild intellectual disability, which may be related to the deletion encompassing *WT1* and other genes in the region, some of which are relevant to neurodevelopment, warranting further follow-up evaluation.

The inheritance patterns of the three families differed, necessitating tailored genetic counseling strategies. Families 1 and 3 involved *de novo* variants, conferring a 50% risk to the offspring of the probands and a low recurrence risk for the parents (with the caveat of excluding gonadal mosaicism). Family 2 exhibited autosomal dominant inheritance (maternal), with a 50% risk to the offspring of carriers. For Families 1 and 3, given the tumor risk associated with WAGR syndrome, multidisciplinary management is recommended, including regular follow-up by ophthalmology, nephrology, oncology, and neurodevelopmental services.

Prenatal diagnosis is an essential component of genetic counseling for families with *PAX6* pathogenic variants. In this study, the mother of Family 2 (2.2) was a heterozygous carrier of c.1234del; upon a subsequent pregnancy, amniocentesis was performed to obtain amniotic fluid samples for prenatal diagnosis. Sanger sequencing revealed that the fetus (2.4) did not carry the variant, indicating a low risk of fetal involvement and providing an important basis for the family's reproductive decision-making. *PAX6*-related aniridia follows autosomal dominant inheritance with high penetrance; for families with an identified pathogenic variant, chorionic villus sampling at 10–13 weeks of gestation or amniocentesis at 16–22 weeks of gestation can be performed to obtain fetal DNA for targeted Sanger sequencing of the known familial variant, enabling rapid and accurate prenatal molecular diagnosis. For families with *de novo* variants (such as Families 1 and 3), although the recurrence risk for parents is low, the possibility of gonadal mosaicism must be excluded, and prenatal diagnosis may still be considered when appropriate.

## Conclusions

Through multi-technique combined detection, three distinct types of pathogenic or likely pathogenic *PAX6* variants were identified in three families with congenital aniridia: a ∼3.19 Mb heterozygous deletion at 11p13 (Family 1, *de novo*), a frameshift variant c.1234del (p.Ser412ProfsTer127; NM_001368894.2) (Family 2, maternal), and a ∼2.998 Mb *de novo* large deletion at 11p14.1p13 (Family 3, *de novo*). All three variants impair *PAX6* function, through haploinsufficiency for the two deletions and disruption of the PST transactivation domain for the frameshift variant. The deletion regions in Families 1 and 3 both encompass the *WT1* gene, meeting the diagnostic criteria for WAGR syndrome, with the proband of Family 3 having been diagnosed with Wilms tumor prior to presentation. This study enriches the variant spectrum of *PAX6*-related aniridia and highlights the important value of combining aCGH, WES, and Sanger sequencing in the precise diagnosis and genetic counseling of aniridia.

## Data Availability

The original contributions presented in the study are included in the article/Supplementary Material, further inquiries can be directed to the corresponding authors.

## References

[1] Vasilyeva TA Marakhonov AV Voskresenskaya AA Kadyshev VV Käsmann-Kellner B Sukhanova NV. Analysis of genotype-phenotype correlations in *PAX6*-associated aniridia. J Med Genet. (2020) 58:270–4. 10.1136/jmedgenet-2019-106172 32467297

[2] Landsend E Lagali N Utheim TP. Congenital aniridia—a comprehensive review of clinical features and therapeutic approach. Surv Ophthalmol. (2021) 66:1031–50. 10.1016/j.survophthal.2021.02.011 33675823

[3] Kit V Cunha DL Hagag AM Moosajee M. Longitudinal genotype-phenotype analysis in 86 patients with *PAX6*-related aniridia. JCI Insight. (2021) 6:e148406. 10.1172/jci.insight.148406 34101622 PMC8410060

[4] Cvekl A Callaerts P. *PAX6*: 25th anniversary and more to learn. Exp Eye Res. (2017) 156:10–21. 10.1016/j.exer.2016.04.017 27126352

[5] Cunha DL Arno G Cortón M Moosajee M. The spectrum of *PAX6* mutations and genotype-phenotype correlations in the eye. Genes (Basel). (2019) 10:1050. 10.3390/genes10121050 31861090 PMC6947179

[6] Vincent M Pujo AL Olivier D Calvas P. Screening for *PAX6* gene mutations is consistent with haploinsufficiency as the main mechanism leading to various ocular defects. Eur J Hum Genet. (2003) 11:163–9. 10.1038/sj.ejhg.5200940 12634864

[7] George AM Duki BT Katz ZS Viswanathan A MacFarland SP Hathaway ER. High-resolution genomic characterization of WAGR spectrum disorder: insights from a novel cohort and literature synthesis, and validation of patient-reported data. Am J Med Genet A. (2026) 194:e70159. 10.1002/ajmg.a.70159 41968606

[8] Wang L Xu Q Wang W Sun X Chen Y. Genetic analysis using next-generation sequencing and multiplex ligation probe amplification in Chinese aniridia patients. Orphanet J Rare Dis. (2024) 19:388. 10.1186/s13023-024-03388-3 39449022 PMC11515619

[9] Zhang R Linpeng S Wei X Li H Huang Y Guo J. Novel variants in *PAX6* gene caused congenital aniridia in two Chinese families. Eye (Lond). (2017) 31:956–61. 10.1038/eye.2016.326 28157223 PMC5518831

[10] Lin ZB Feng CY Li J Pan AP Sun HS Yu AY. A rare missense *PAX6* mutation causes atypical aniridia in a three-generation Chinese family. Int J Ophthalmol. (2024) 17:466–72. 10.18240/ijo.2024.03.07 38721508 PMC11074177

[11] Riggs ER Andersen EF Cherry AM Kantarci S Kearney H Patel A. Technical standards for the interpretation and reporting of constitutional copy-number variants: a joint consensus recommendation of the American college of medical genetics and genomics (ACMG) and the clinical genome resource (ClinGen). Genet Med. (2020) 22:245–57. 10.1038/s41436-019-0686-8 31690835 PMC7313390

[12] Richards S Aziz N Bale S Bick D Das S Gastier-Foster J. Standards and guidelines for the interpretation of sequence variants: a joint consensus recommendation of the American college of medical genetics and genomics and the association for molecular pathology. Genet Med. (2015) 17:405–24. 10.1038/gim.2015.30 25741868 PMC4544753

[13] Yang SH He H Hou S Yang T Yin Z Zhang H. ML-ExonCNV: a robust XGBoost multi-expert ensemble framework for rare exon CNV detection in whole-exome sequencing data. Brief Bioinform. (2026) 27(2):bbag100. 10.1093/bib/bbag100 41818723 PMC12981628

[14] Abou Tayoun AN Pesaran T DiStefano MT Oza A Rehm HL Biesecker LG. Clingen sequence variant interpretation working group. Recommendations for interpreting the loss of function PVS1 ACMG/AMP variant criterion. Hum Mutat. (2018) 39:1517–25. 10.1002/humu.23626 30192042 PMC6185798

[15] Tavtigian SV Greenblatt MS Harrison SM Nussbaum RL Prabhu SA Boucher KM. Clingen sequence variant interpretation working group. Modeling the ACMG/AMP variant classification guidelines as a Bayesian classification framework. Genet Med. (2020) 22:1054–60. 10.1038/gim.2017.210 PMC6336098 29300386

[16] Wang C Yang W Li X Zhou C Liu J Jin L. A novel *PAX6* frameshift mutation identified in a large Chinese family with congenital aniridia. J Pers Med. (2023) 13:442. 10.3390/jpm13030442 36983625 PMC10052173

[17] Hu P Meng L Ma D Qiao F Wang Y Zhou J. A novel 11p13 microdeletion encompassing *PAX6* in a Chinese Han family with aniridia, ptosis and mental retardation. Mol Cytogenet. (2015) 8:27. 10.1186/s13039-015-0110-2 25628759 PMC4307215

[18] Van Heyningen V Hoovers JMN De Kraker J Crolla JA. Raised risk of wilms tumour in patients with aniridia and submicroscopic *WT1* deletion. J Med Genet. (2007) 44:787–90. 10.1136/jmg.2007.051318 17630404 PMC2652818

[19] Li Y Chen J Zheng Y Chen Z Wang T Sun Q. A novel microdeletion of 517 kb downstream of the *PAX6* gene in a Chinese family with congenital aniridia. BMC Ophthalmol. (2023) 23:399. doi: 10.1186/s12886-023-03147-1\ 37752489 10.1186/s12886-023-03147-1 PMC10523764

