# Supplementary material for: Diverse PAX6 variants in three Chinese families with congenital aniridia: genotype–phenotype correlations and diagnostic implications
Source: Front Med (Lausanne). 2026 Oct 21;13:1978944. doi: 10.3389/fmed.2026.1978944 (PMC13730820; doi:10.3389/fmed.2026.1978944)
Supplement: Supplementary file 1 [file Datasheet1.zip › Supplementary files/Table S1 PCR Primers and amplification conditions for qRT-RCR.docx]

**Table S1. PCR Primers for** **qRT-PCR**

| **Primers** | **Sequence** | **Tm** |
| --- | --- | --- |
| *PAX6*-exon2-F | AGCCATCACCAATCAGCATAGG | 60℃ |
| *PAX6*-exon2-R | GGTTGGTGTGTGAGAGCAATTC |  |
| *PAX6*-exon7-F | AGGGGGTCTGTACCAACGATAA | 60℃ |
| *PAX6*-exon7-R | GTCAGGGCATTCCTCTCTGTTC |  |
| *PAX6*-exon14-F | GCTGTTAGAGCCGCTTCAGT | 60℃ |
| *PAX6*-exon14-R | GTTCCAACTGATATCGTGCCTTC |  |
| β-globin-QF | ACACAACTGTGTTCACTAGC | 60℃ |
| β-globin-QR | CAACTTCATCCACGTTCACC |  |

**Reaction reagents and condition**

The expression level was assessed by real-time Quantitative Fluorescence PCR using SYBR Premis Ex Taq II(Perfect Real Time) (Takara) with ABI 7500 system. Data are presented as mean±standard deviation of three independent real-time PCR experiments. The PCR cycle was as follows: 10 min 95°C, 1 cycle; 10 s 95°C, 30 s 60°C＋fluorescence acquisition, 55 cycles. Values for each gene were normalized to expression level of beta-actin gene (ACTB) via the 2^-ΔΔCT^ method.
