# Supplementary material for: Diverse PAX6 variants in three Chinese families with congenital aniridia: genotype–phenotype correlations and diagnostic implications
Source: Front Med (Lausanne). 2026 Oct 21;13:1978944. doi: 10.3389/fmed.2026.1978944 (PMC13730820; doi:10.3389/fmed.2026.1978944)
Supplement: Supplementary file 1 [file Datasheet1.zip › Supplementary files/Table S2 PCR Primers and amplification conditions for DNA.docx]

**Table S2 PCR Primers and amplification conditions for DNA**

| **Primers** | **Sequence** | **Tm** | **PCR enzyme and buffer** |
| --- | --- | --- | --- |
| PAX6-exon13-F | TGATCAGACTTGTTGGCAGAG | TD | PrimeSTAR® HS DNA Polymerase |
| PAX6-exon13-R | AAGGAGTTAAACACGCCCTC |  |  |

TD, Touch-down protocol; ­­­

**Touch-down** protocol comprised of initial step of 95°C for 5 min, followed by 21 cycles of 95°C for 30 sec, 70°C for 30 seconds with decreasing 1°C per cycle, and 72°C for 30 sec, then followed by 25 cycles of 95°C for 30 sec, 50°C for 30 sec, and 72°C for 30 sec, with a final extension step of 72°C for 10 mins.
