# Supplementary material for: Diverse PAX6 variants in three Chinese families with congenital aniridia: genotype–phenotype correlations and diagnostic implications
Source: Front Med (Lausanne). 2026 Oct 21;13:1978944. doi: 10.3389/fmed.2026.1978944 (PMC13730820; doi:10.3389/fmed.2026.1978944)
Supplement: Supplementary file 2 [file Table1.docx]

**Supplementary Material**

Manuscript: Diverse *PAX6* variants in three Chinese families with congenital aniridia: genotype–phenotype correlations and diagnostic implications

Supplementary Tables S1 and S2 (qPCR and Sanger sequencing primer sequences) are provided in the original submission. This file contains Supplementary Table S3.

**Supplementary Table S3. Reported *PAX6* variants in patients with congenital aniridia**

| **No.** | **Reference** | **Population** | **cDNA variant** | **Protein change** | **Variant type** | **Exon/domain** | **Phenotype** | **Detection method** | **Inheritance** |
| --- | --- | --- | --- | --- | --- | --- | --- | --- | --- |
| 1 | This study (Family 1) | Chinese | arr[GRCh37] 11p13(31,037,164_34,227,327)×1 (~3.19 Mb), encompassing *PAX6* and *WT1* | — | CNV (deletion) | 11p13; *PAX6* + *WT1* | Aniridia; WAGR risk (*WT1* co-deleted) | aCGH + qPCR | De novo |
| 2 | This study (Family 2) | Chinese | c.1234del (NM_001368894.2) | p.Ser412ProfsTer127 | Frameshift (C-terminal extension) | Exon 14/PST | Aniridia, lens opacities, nystagmus | WES + Sanger sequencing | AD (maternal) |
| 3 | This study (Family 3) | Chinese | ~2.998 Mb del(11)(p14.1p13) NC_000011.9:g.30352495_33350179del, encompassing *PAX6* and *WT1* | — | CNV (deletion) | 11p14.1p13; *PAX6* + *WT1* | Aniridia, foveal hypoplasia, Wilms tumor, mild intellectual disability; WAGR | WES read-depth (ML-ExonCNV v1.0.0) + qPCR | De novo |
| 4 | S1 | Chinese | c.28C>T | p.Gln10Ter | Nonsense | Exon 5/PD | Aniridia | Panel NGS + Sanger sequencing | AD/familial |
| 5 | S1 | Chinese | c.607C>T | p.Arg203Ter | Nonsense | Exon 8/LNK | Aniridia | Panel NGS + Sanger sequencing | AD/familial |
| 6 | S1 | Chinese | c.812_813del | p.Leu271Glyfs*12 | Frameshift | Exon 10/PST | Aniridia | Panel/targeted NGS + Sanger sequencing | AD/familial |
| 7 | S1 | Chinese | c.1268A>T | p.Ter423Leu (stop-loss; C-terminal extension) | Nonsense (stop-loss) | Exon 13/PST | Aniridia | Panel NGS + Sanger sequencing | AD/familial |
| 8 | S1 | Chinese | Whole-gene or adjacent-gene deletions of *PAX6* (12 families) | — | CNV (deletion) | 11p13 | Aniridia (2 patients with WAGR spectrum features) | MLPA | AD/familial |
| 9 | S2 | Chinese | c.50dupA | p.Asn17Lysfs*39 | Frameshift | Exon 5/PD | Aniridia, cataract, nystagmus | Sanger sequencing + RT-qPCR | AD |
| 10 | S2 | Chinese | c.765+1_765+2delGT | Exon 9 skipping (p.E228Gfs*5) | Splice-site | Intron 9 | Aniridia, cataract, nystagmus | Sanger sequencing + RT-qPCR | AD |
| 11 | S3 | Chinese | c.114_119delinsAATTTC | p.Pro39IlefsTer17 | Frameshift (delins) | Exon 5/PD | Aniridia, cataract, glaucoma, foveal hypoplasia | WES + Sanger sequencing | AD |
| 12 | S4 | Chinese | c.391_398dupATACCAAG (NM_001604) | p.Ser133Argfs*8 | Frameshift | Exon 7 | Aniridia, foveal hypoplasia, nystagmus | Targeted NGS + Sanger sequencing | AD |
| 13 | S5 | Chinese | 517 kb deletion downstream of *PAX6* (chr11:31,139,019-31,655,997, GRCh38), 3′ regulatory enhancers | — | CNV (regional deletion) | 11p13, downstream of *PAX6* | Aniridia, nystagmus (mild phenotype) | WES (XHMM v1.0 + CNVkit) + qPCR + long-range PCR | AD |
| 14 | S6 | Chinese | 518 kb microdeletion 11p13 encompassing *PAX6*, *ELP4*, and partial *IMMP1L* | — | CNV (deletion) | 11p13 | Aniridia, ptosis, intellectual disability | SNP-array + MLPA | AD |
| 15 | S7 | Japanese | 474delC (as published; legacy numbering) | — | Frameshift | Exon 5/paired domain | Aniridia (mild) | Direct DNA sequencing (Sanger) | AD (familial) |
| 16 | S7 | Japanese | 678_688del11 (as published; legacy numbering) | — | Frameshift | Exon 6/paired domain | Aniridia | Direct DNA sequencing (Sanger) | Sporadic |
| 17 | S7 | Japanese | 572_575delAATCins14 (as published; legacy numbering) | — | Frameshift (indel) | Exon 6/paired domain | Aniridia; VATER association in one sib | Direct DNA sequencing (Sanger) | AD (familial) |
| 18 | S7 | Japanese | 786_787ins10 (10-bp duplication, as published; legacy numbering) | — | Frameshift | Exon 7/linker region | Aniridia | Direct DNA sequencing (Sanger) | AD (familial) |
| 19 | S8 | Japanese | c.607C>T | p.Arg203Ter | Nonsense | Exon 8/LNK | Aniridia (variable iris phenotype between two patients) | PCR + direct sequencing | Sporadic |
| 20 | S8 | Japanese | c.718C>T | p.Arg240Ter | Nonsense | Exon 9/HD | Aniridia | PCR + direct sequencing | AD (familial) and sporadic |
| 21 | S8 | Japanese | c.131G>A | p.Arg44Gln | Missense | Exon 5/PD (N-terminal subdomain) | Aniridia | PCR + direct sequencing | Sporadic |
| 22 | S9 | Danish | 985G>A (R208Q, as published; legacy numbering) | p.Arg208Gln (legacy numbering) | Missense | Exon 8/linker region | Aniridia (very mild); non-penetrant in carrier mother | Dideoxy fingerprinting + DNA sequencing | AD (familial) |
| 23 | S9 | Danish | 1311C>T (R317X, as published; legacy numbering) | p.Arg317Ter (legacy numbering) | Nonsense | Exon 11/PST | Aniridia | Dideoxy fingerprinting + DNA sequencing | AD (familial) |
| 24 | S9 | Danish | Frameshift in exon 12 from codon 386 (as published; legacy numbering) | C-terminally extended protein (longer than wild type) | Frameshift (C-terminal extension) | Exon 12/PST | Aniridia (moderately mild) | Dideoxy fingerprinting + DNA sequencing | Not determined |
| 25 | S10 | British (UK) | c.345_351dupTAACATA (NM_000280.4) | p.Pro118* | Frameshift (novel) | Paired domain | Bilateral complete iris hypoplasia, foveal hypoplasia, cataract | Sanger sequencing | Sporadic |
| 26 | S10 | British (UK) | c.2T>G (NM_000280.4) | p.Met1Arg (start-codon variant) | Start-codon (missense class) | Exon 4/start codon | Aniridia (severe iris hypoplasia) | Sanger sequencing | Not stated |
| 27 | S10 | British (UK) | Deletion of the *PAX6* 3′ regulatory region extending into *ELP4* and *DCDC1* | — | CNV (regulatory deletion) | 11p13, 3′ of *PAX6* | Partial iris hypoplasia, cataract, glaucoma, retinal detachment (no keratopathy) | Genetic screening (diagnostic laboratory) | Not stated |

AD, autosomal dominant; aCGH, array comparative genomic hybridization; CNV, copy number variant; HD, homeodomain; LNK, linker region; MLPA, multiplex ligation-dependent probe amplification; NMD, nonsense-mediated mRNA decay; PD, paired domain; PST, proline-serine-threonine-rich (transactivation) domain; qPCR, quantitative real-time polymerase chain reaction; VATER, vertebral defects, anal atresia, tracheoesophageal fistula, radial dysplasia, and renal anomalies; WES, whole-exome sequencing; WAGR, Wilms tumor, aniridia, genitourinary anomalies, and intellectual disability.

Note: Variant nomenclature is given as published. Most studies used transcript NM_000280.6 of *PAX6* (or earlier versions); the studies by Grønskov et al. (S9) and Kawano et al. (S7) predated current transcript-based nomenclature and used legacy numbering, as indicated in the table. Protein descriptions for the same cDNA position may differ between studies because of differences in the application of the HGVS 3′ rule. The current MANE Select transcript NM_001368894.2 contains an additional alternatively spliced exon (exon 5a, 42 bp); cDNA positions 3′ of exon 5a differ by 42 nucleotides between the two transcripts (e.g., NM_000280.6:c.1192del corresponds to NM_001368894.2:c.1234del). The Population column indicates the population/ethnicity of the cohort in which the variant was reported, as stated in the source publication. Phenotypes are abbreviated; full clinical descriptions are provided in the referenced studies.

**Supplementary references**

S1. Wang L, Xu Q, Wang W, Sun X, Chen Y. Genetic analysis using next-generation sequencing and multiplex ligation probe amplification in Chinese aniridia patients. Orphanet J Rare Dis (2024) 19:388. doi: 10.1186/s13023-024-03388-3.

S2. Zhang R, Linpeng S, Wei X, Li H, Huang Y, Guo J, et al. Novel variants in PAX6 gene caused congenital aniridia in two Chinese families. Eye (Lond) (2017) 31:956-961. doi: 10.1038/eye.2016.326.

S3. Ouyang J, Cai Z, Guo Y, Nie F, Cao M, Duan X. Detection of a novel PAX6 variant in a Chinese family with multiple ocular abnormalities. BMC Ophthalmol (2022). doi: 10.1186/s12886-022-02256-7.

S4. Wang C, Yang W, Li XM, Zhou C, Liu J, Jin L, et al. A novel PAX6 frameshift mutation identified in a large Chinese family with congenital aniridia. J Pers Med (2023) 13:442. doi: 10.3390/jpm13030442.

S5. Li Y, Chen J, Zheng Y, Chen Z, Wang T, Sun Q, et al. A novel microdeletion of 517 kb downstream of the PAX6 gene in a Chinese family with congenital aniridia. BMC Ophthalmol (2023) 23:399. doi: 10.1186/s12886-023-03147-1.

S6. Hu P, Meng L, Ma D, Qiao F, Wang Y, Zhou J, et al. A novel 11p13 microdeletion encompassing PAX6 in a Chinese Han family with aniridia, ptosis and mental retardation. Mol Cytogenet (2015) 8:27. doi: 10.1186/s13039-015-0110-2.

S7. Kawano T, Wang C, Hotta Y, Sato M, Iwata-Amano E, Hikoya A, et al. Three novel mutations of the PAX6 gene in Japanese aniridia patients. J Hum Genet (2007). doi: 10.1007/s10038-007-0153-2.

S8. Yokoi T, Nishina S, Fukami M, Ogata T, Hosono K, Hotta Y, et al. Genotype–phenotype correlation of PAX6 gene mutations in aniridia. Hum Genome Var (2016). doi: 10.1038/hgv.2015.52.

S9. Grønskov K, Rosenberg T, Sand A, Brøndum-Nielsen K. Mutational analysis of PAX6: 16 novel mutations including 5 missense mutations with a mild aniridia phenotype. Eur J Hum Genet (1999). doi: 10.1038/sj.ejhg.5200308.

S10. Kit V, Lima Cunha D, Hagag AM, Moosajee M. Longitudinal genotype-phenotype analysis in 86 patients with PAX6-related aniridia. JCI Insight (2021) 6:e148406. doi: 10.1172/jci.insight.148406.
